# Detection of Multiple Transgene Fragments in a Mouse Model of Gene Doping Based on Plasmid Vector Using TaqMan-qPCR Assay

**DOI:** 10.3390/genes11070750

**Published:** 2020-07-06

**Authors:** Takehito Sugasawa, Kai Aoki, Kouki Yanazawa, Kazuhiro Takekoshi

**Affiliations:** 1 Laboratory of Laboratory/Sports Medicine, Division of Clinical Medicine, Faculty of Medicine, University of Tsukuba, 1-1-1 Tennodai, Tsukuba 305-8577, Ibaraki, Japan; take0716@krf.biglobe.ne.jp; 2 Doctoral Program in Sports Medicine, Graduate School of Comprehensive Human Sciences, University of Tsukuba, 1-1-1 Tennodai, Tsukuba 305-8577, Ibaraki, Japan; fineday0126@gmail.com; 3 Master’s Program in Medical Sciences, Graduate School of Comprehensive Human Sciences, University of Tsukuba, 1-1-1 Tennodai, Tsukuba 305-8577, Ibaraki, Japan; s1921312@s.tsukuba.ac.jp

**Keywords:** gene doping, gene therapy, in vivo transfection, in vivo imaging

## Abstract

The World Anti-Doping Agency has prohibited gene doping in the context of progress in gene therapy. There is a risk that the augmentation of genes using plasmids could be applied for gene doping. However, no gold standard method to detect this has been established. Here, we aimed to develop a method to detect multiple transgene fragments as proof of gene doping. Firstly, gene delivery model mice as a mimic of gene doping were created by injecting firefly luciferase plasmid with polyethylenimine (PEI) into the abdominal cavity. The results confirmed successful establishment of the model, with sufficient luminescence upon in vivo imaging. Next, multiple transgene fragments in the model were detected in plasma cell-free (cf)DNA, blood-cell-fraction DNA, and stool DNA using the TaqMan- quantitative real-time PCR(qPCR) assay, with the highest levels in plasma cfDNA. Using just a single drop of whole blood from the model, we also attempted long-term detection. The results showed that multiple transgene fragments were detected until 11 days. These findings indicate that the combination of plasma cfDNA or just one drop of whole blood with TaqMan-qPCR assay is feasible to detect plasmid-PEI-based gene doping. Our findings could accelerate the development of methods for detecting gene doping in humans.

## 1. Introduction

Doping is the act of raising competitive abilities to achieve success in sports by using prohibited substances or methods [1]. Doping in sports, especially at events such as the Olympic Games and at national, regional, or world championships, is considered illegal and against the spirit of good sportsmanship. The World Anti-Doping Agency (WADA), which was established in 1999, is involved in scientific research on doping, anti-doping education, development of anti-doping strategies, and monitoring of the World Anti-Doping Code [2] to ensure soundness and fairness in sports worldwide. However, despite WADA’s substantial efforts, doping has not been eradicated.

For example, it was recently announced that WADA’s Executive Committee unanimously endorsed a 4-year ban for non-compliance by the Russian Anti-Doping Agency, following falsification of data related to Russian doping fraud [3]. As a result, it will be virtually impossible for Russian sports teams to participate in major international competitions including the 2020 Tokyo Olympics and Paralympics and the 2022 Football World Cup in Qatar. As accurate testing of doping in athletes’ samples is extremely important to eliminate such doping violations, researchers working in support of WADA develop new and accurate examination methods every year.

Recently, within the medical field, there has been accelerated progress in gene therapy as a new therapeutic strategy with the potential to treat a range of inherited and acquired diseases [4]. In fact, several gene therapy drugs, such as Collategen [5] (AnGes, Inc., Osaka, Japan), Zolgensma [6] (AveXis, Inc., Chicago, IL, USA), and YESCARTA [7] (Kite Pharma, Inc., Santa Monica, CA, USA), have been approved for treating human diseases on prescription. Simultaneously, the number of clinical trials of gene therapy has been increasing year by year [4,8]. However, this has raised concerns at WADA because the historical background shows that doping has evolved along with the progression of medical treatment. From an early stage, WADA added gene doping to its list of prohibited actions. Subsequently, in January 2018, WADA extended the ban on gene doping to include any forms of gene regulation. Moreover, in January 2020, WADA once again modified its rules to be in line with current progress in gene editing and gene delivery technology for gene therapy. The prohibited gene doping methods are currently described in detail as follows: “The use of nucleic acids or nucleic acid analogues that may alter genome sequences and/or alter gene expression by any mechanism” [9]. However, there is as yet no established gold standard method to detect gene doping in human specimens.

Recently, we have focused on plasmid with polyethylenimine (PEI; cationic polymer) as a tool for gene delivery with high transfection efficacy because plasmids are likely to be used as vectors for gene doping. There have been many studies about gene therapy based on animal experiments using plasmid-PEI complex [10,11,12,13,14], which can increase the efficiency of gene delivery to cells. A search of ClinicalTrials.gov [15], a database of privately and publicly funded clinical studies, revealed three clinical trials using plasmid-PEI complex since 2011, including two ongoing trials. It is thus also possible that plasmid-PEI complex could be used for gene doping in human athletes. However, there is currently no established gold standard method for detecting gene doping using plasmid-PEI complex in humans.

Here, we first aimed to create gene delivery model mice using plasmid-PEI complex, as a mimic of gene doping. We also aimed to discover suitable specimens and to develop a method for detecting multiple transgene fragments, as proof of gene doping, in the model mice.

## 2. Materials and Methods

### 2.1. Animals

**Figure 1:**
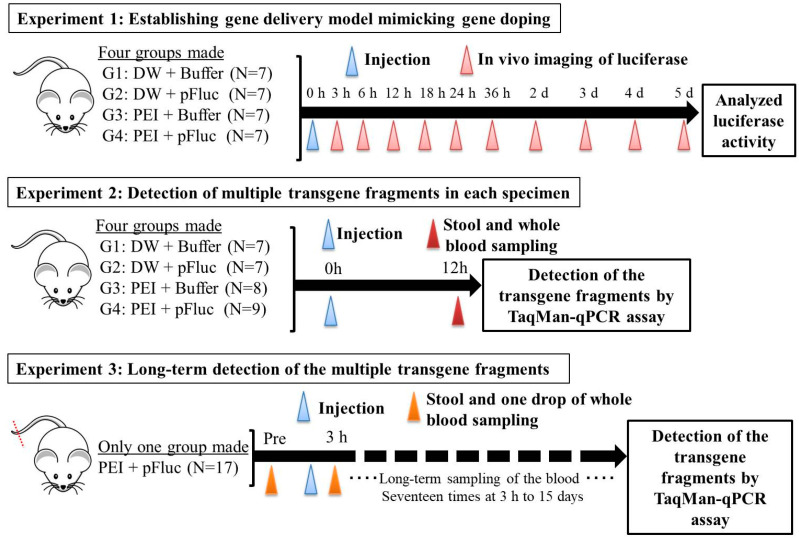
Overview of the experimental protocols in this study. DW: distilled water, PEI: polyethylenimine, pFluc: firefly luciferase plasmid, G: group.

All animal experiments in this study were approved by the Animal Care Committee, University of Tsukuba (approval numbers: 19–163 and 19–425). Seven-week-old ICR male mice were purchased from the Central Laboratories for Experimental Animals (Tokyo, Japan) and then subjected to a 1 week acclimation period. The mice were bred and maintained in an air-conditioned animal house under specific-pathogen-free conditions and subjected to a 12/12 h light and dark cycle. The mice were fed standard mouse pellets and water ad libitum. At the start of the experiments, the weights and age of the mice were 35.2–43.4 g and 8 weeks. An overview of the animal protocols of these experiments is shown in Figure 1.

### 2.2. Experiment 1: Establishing the Gene Delivery Model Mimicking Gene Doping

Here, we established a gene delivery model in mice using pSF-CMV-Fluc plasmid (cytomegalovirus promoter-firefly luciferase plasmid; hereafter, “pFluc”; OXGENE, Oxford, UK) with PEI MAX (hereafter, “PEI”; PolySciences, Warrington, PA, USA), which can mimic gene doping. The pFluc-PEI complex enabling in vivo transfection was created by mixing the following solutions: 75 μL of PEI (4 mg/mL) solved in distilled water (DW)/100 μL of pFluc (1 μg/μL) solved in Tris-EDTA (TE) buffer (pH 7.4)/100 μL of 25% glucose solution/225 μL of DW, for a total volume of 500 μL per mouse. The ratio of DNA:PEI as weight was 1:3. After making the mixtures, they were incubated for 10 min at room temperature. Next, in vivo transfection was performed by injecting each mixture into the abdominal cavity. The mice that received in vivo transfection of pFluc with PEI were assigned to group 4 (G4; PEI + pFluc; *n* = 7). Three control groups were also established by injecting 500 μL of each solution as follows: group 1 (G1; DW + Buffer; *n* = 7), group 2 (G2; DW + pFluc; *n* = 7), and group 3 (G3; PEI + Buffer; *n* = 7). At each of the time points of 3, 6, and 12 h and 1, 2, 3, 4, and 5 days after the injection, the mice were anesthetized by isoflurane inhalation, and then injected with phosphate-buffered saline (PBS) solution in which D-luciferin (Cayman, Ann Arbor, MI, USA) at 75 mg/kg was solved into the abdominal cavity. At 5 min after the injection, luminescence, as an indicator of gene delivery, was measured across the whole body under anesthesia using IVIS Imaging System (IVIS Spectrum; PerkinElmer, Waltham, MA, USA) with the following settings: imaging mode: luminescent, exposure time: 60 s, binning: medium, F/stop: 1, emission filter: open, and field of view: D. Black and white photos of the mice were also taken at the same time to overlie the luminescence photos with the following settings: imaging mode: photograph, binning: medium, and F/stop: 8. Then, the luminescence levels were quantified as averaged counts using Living Image Software (PerkinElmer, Waltham, MA, USA).

### 2.3. Experiment 2: Detection of Multiple Transgene Fragments in Each Specimen

In the experiment described in the previous section, because the maximum luminescence reflecting gene transfer was observed at 12 h after injection, we changed the time of detection of the multiple transgene fragments of the plasmid in each specimen to this time point.

In this experiment, the same four groups (G1: *n* = 7, G2: *n* = 7, G3: *n* = 8, and G4: *n* = 9) were again established as in experiment 1, and the injections of the same mixtures were performed. At 12 h after injection, stool samples were harvested from the mice placed in an empty cage and then quickly transferred on ice. Next, the mice were sacrificed under general anesthesia using isoflurane inhalation followed by whole blood collection with EDTA-2Na (ethylenediaminetetraacetic acid-disodium salt). The whole blood samples were centrifuged at 3000 rpm for 10 min at 4 °C and then aliquots of the plasma fraction were collected. The blood cell fractions including white and red blood cells were also collected. These specimens were stored at −20 °C until further analysis.

Total DNA was extracted from collected stool, plasma, and blood cell fractions. A phenol/chloroform/isoamyl alcohol solution (Nacalai Tesque, Kyoto, Japan) was used to extract total DNA from the stool in accordance with the manufacturer’s instructions. Using 200 μL of the plasma, cell-free (cf)DNA was extracted using NucleoSpin Plasma XS (Takara Bio, Kusatsu, Japan) kit at a final elution volume of 30 μL, in accordance with the manufacturer’s instructions. Using 100 μL of the blood cell fraction, total DNA was extracted using NucleoSpin Blood QuickPure (Takara Bio, Kusatsu, Japan) kit at a final elution volume of 50 μL, in accordance with the manufacturer’s instructions. The concentrations of DNA were measured using extracted DNA solutions of stool and blood cell fractions on NanoDrop 1000 (Thermo Fisher Scientific, Waltham, MA, USA). Then, the concentrations were adjusted to 10 ng/μL for all samples for further analyses. For the plasma cfDNA, the concentrations could not be measured because they were very low. Therefore, undiluted solutions of plasma cfDNA were used for further analyses.

To perform TaqMan-quantitative real-time PCR (qPCR) assay to detect multiple transgene fragments, the primers and TaqMan probes were designed with targeting firefly luciferase gene (Fluc), ampicillin resistance gene (Amp), and cytomegalovirus promoter (CMVp) for the specific amplification of the plasmid sequences, using Primer-BLAST (NIH National Library of Medicine, Bethesda, MD, USA). The primers and probes were also checked for specificity by in silico PCR using Primer-BLAST; this confirmed that there was no amplification from the human and mouse genomes. The sequences of the primers and TaqMan probes are shown in Table 1. The primers and probes as a double quencher system were systemized by Integrated DNA Technologies (Coralville, IA, USA).

Next, TaqMan-qPCR assay was performed to detect multiple transgene fragments as absolute quantification using PrimeTime Gene Expression Master Mix (Integrated DNA Technologies, Coralville, IA, USA) with the primers and TaqMan probes on QuantStudio 5 Real-Time PCR Systems (Thermo Fisher Scientific) as duplicated measurement. The template volume, and primer and probe concentrations were 2 µL, 100 nM, and 100 nM, respectively, for a total reaction volume of 10 µL per well. pFluc was used at 100 pg/μL to make a standard curve for absolute quantification, and the range of the standard curve could be set to 1.56 × 10^8^ to 2.49 copies/μL on the three primers-probes. Negative control wells were also established using pure water. The conditions of thermal cycling were 95 °C for 5 min, followed by 40 cycles of 95 °C for 2 s and 60 °C for 20 s. All standard curves had *R*^2^ > 0.98.

### 2.4. Experiment 3: Long-Term Detection of Multiple Transgene Fragments

In the experiment described in the previous section, as just a single drop of whole blood could be a specimen enabling highly sensitive detection of transgene fragments, we performed the following experiment using one drop of blood and explored the temporal detection limit in each mouse. Simultaneously, we also collected the stools as auxiliary specimens.

Using 17 untreated ICR mice, about 2 mm of the tail tip was cut, after which about 50 μL of whole blood, just a single drop as a pre-experiment specimen, was collected into a 1.5-mL microtube including 150 μL of PBS-EDTA 2Na mixture. Then, the collected blood was quickly put on ice and stored at −20 °C until further analysis. Simultaneously, the stools were also collected using the same method as in experiment 2 and stored the same way as the blood. One week later, the pFluc-PEI complex was injected into the abdominal cavity the same way as mentioned above. Subsequently, one drop of whole blood and stools were collected at 3, 6, 12, 24, 36, 48, and 60 h, and 3, 4, 5, 6, 7, 8, 9, 11, 13, and 15 days after the injection in the same way. Total DNA was extracted from the collected whole blood using NucleoSpin Blood QuickPure and then adjusted to 10 ng/μL the same as in the above-mentioned methods. The total DNA of the stool was also extracted by the same methods as in experiment 2. Using the total DNA, qPCR was performed to detect multiple transgene fragments in the same way as in the above-mentioned methods.

### 2.5. Determination of Specificity of the Primers and TaqMan Probe Specificity

The same DNA samples from each specimen in experiment 2 (Section 2.3) were pooled into one 1.5 mL microtube. Using the pooled DNA of each specimen, qPCR was performed to detect transgene fragments using the same primers and probes in the same way as mentioned above, with a positive control of pFluc (100 pg/μL). After the qPCR, the amplicons were collected in a 1.5 mL microtube, and then were purified using NucleoSpin Gel and PCR Clean-up kit (Takara Bio, Kusatsu, Japan). The purified DNA was subjected to Sanger sequencing to check the sequence of the amplicons via outsourcing to an external company (FASMAC, Kanagawa, Japan). The data from the Sanger sequencing were analyzed on CLC Sequence Viewer ver. 8.0 (QIAGEN, Hilden, Germany) and BioEdit ver. 7.2.5 (developer: Tom Hall).

### 2.6. Statistics

All data were statistically analyzed using GraphPad Prism version 7.04 (San Diego, CA, USA). First, for all experimental data, we conducted the Shapiro-Wilk normality test to check the normality of the distributions. Subsequently, we decided to use non-parametric tests for all data. Kruskal-Wallis H tests (one-way ANOVA of ranks) were also performed, followed by a two-stage Benjamini, Krieger, and Yekutieli False Discovery Rate (FDR) procedure as a post hoc test. A *p* value less than 0.05 was considered to be statistically significant. In all graphs, the *y*-axis is displayed logarithmically.

## 3. Results

### 3.1. Gene Delivery was Successful by Injecting Pfluc-PEI Complex, Particularly after 12 h, in Experiment 1

**Figure 2:**
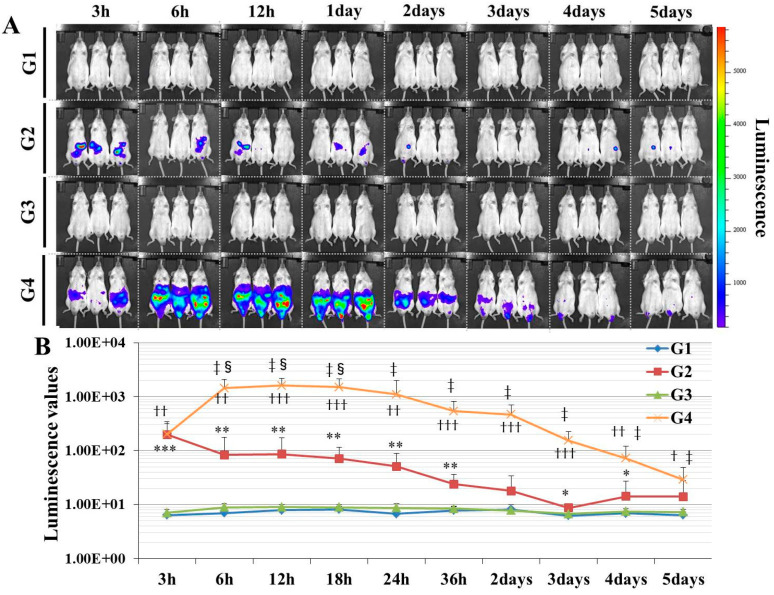
Establishing the gene delivery model mimicking gene doping. (**A**): Chronic in vivo imaging of luminescence of representative mice after the injection of each solution: G1: DW + Buffer (N = 7), G2: DW + pFluc (N = 7), G3: PEI + Buffer (N = 7), and G4: PEI + pFluc (N = 7). The luminescence is shown as a color scale. (**B**): The chronological quantification values of intensity of the luminescence in each group and the data are shown as mean ± SD. The significant differences at each time point are shown for G1 vs. G2 as *: *p <* 0.05, **: *p <* 0.01, and ***: *p* < 0.001; G3 vs. G4 as †: *p* < 0.05, ††: *p* < 0.01, and †††: *p* < 0.001; G2 vs. G4 as ‡: *p* < 0.05; and 3 h vs. 6, 12, or 18 h within G4 as §: *p* < 0.05.

In G1 (DW + Buffer) and G3 (PEI + Buffer), which did not include pFluc, there was no luminescence in the mice at any time point. On the other hand, in G2 (DW + pFluc) and G4 (PEI + pFluc), luminescence indicating successful gene delivery was confirmed at some time points. Additionally, G4 showed higher luminescence than G2 at each time point. The highest luminescence was confirmed in G4 at 12 h after injection. In G4, long-term luminescence was also confirmed over 5 days (Figure 2).

### 3.2. Multiple Transgene Fragments were Detected in Each Specimen in Experiment 2

**Figure 3:**
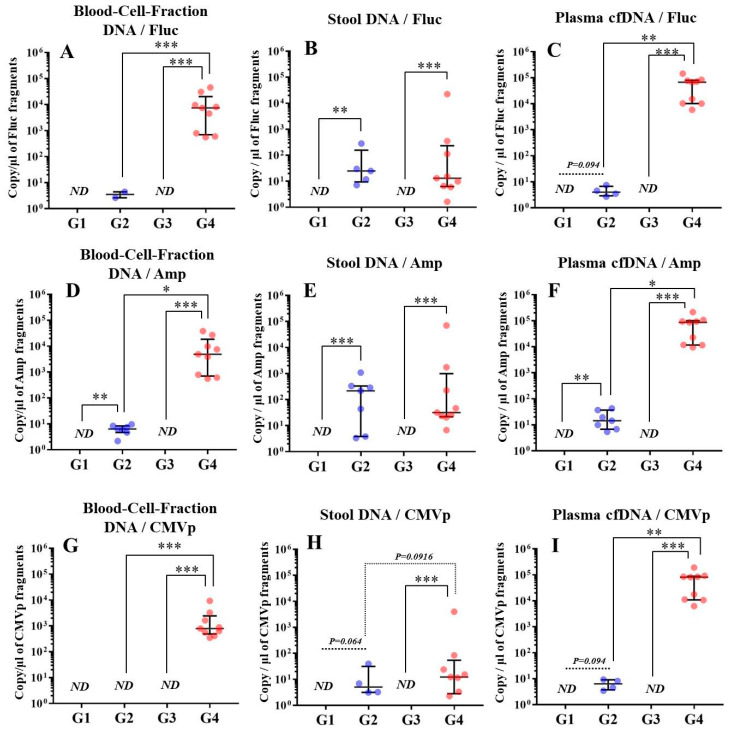
Detection of multiple transgene fragments in each specimen.(**A**–**C**) show the results using Fluc primer-probe in each specimen, (**D**–**F**) show the results using Amp primer-probe in each specimen, and (**G**–**I**) show the results using CMVp primer-probe in each specimen. G1: DW + Buffer (*n* = 7), G2: DW + pFluc (*n* = 7), G3: PEI + Buffer (*n* = 8), and G4: PEI + pFluc (*n* = 9). The individual data are shown as a plot with bars representing the median and interquartile range. ND: not detected. *: *p* < 0.05, **: *p* < 0.01, and ***: *p* < 0.001.

In G1 and G3, no transgene fragments were detected in any specimens. On the other hand, in G4, multiple transgene fragments including Fluc, Amp, and CMVp were detected in all DNA specimens of blood-cell-fraction DNA, stool DNA, and plasma cfDNA. In G2 (DW + pFluc), fewer transgene fragments than in G4 were detected in the blood-cell-fraction DNA and plasma cfDNA. In the stool DNA, the transgene fragments were detected in G2 and G4, showing no difference in the levels between these two groups (Figure 3).

**Figure 4:**
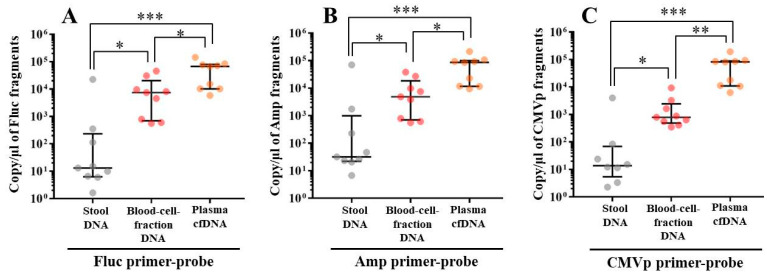
Comparison of the abundance of multiple transgene fragments among stool DNA, blood-cell-fraction DNA, and plasma cfDNA within G4. (**A**): Using the Fluc primer-probe, (**B**): using the Amp primer-probe, and (**C**): using the CMVp-primer probe. The number of samples is nine in each group. The individual data are shown as a plot with bars indicating median and interquartile range. ND: not detected. *: *p* < 0.05, **: *p* < 0.01, and ***: *p* < 0.001.

Upon performing an analysis within G4, the transgene fragments were mostly present in plasma cfDNA (Figure 4). No differences of detection efficacy among these primers-probes were confirmed (Figure 4).

### 3.3. Multiple Transgene Fragments Were Detected until 9 Days in the DNA from One Drop of Blood, but with Lower Sensitivity for Stool DNA

**Figure 5:**
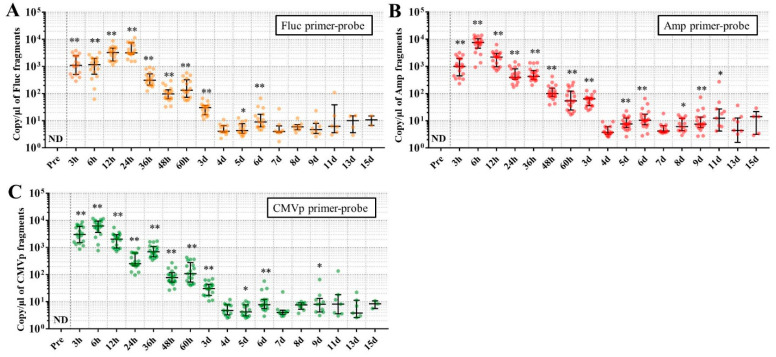
Long-term detection of multiple transgene fragments in the DNA from one drop of blood. (**A**): The results using Fluc primer-probe; (**B**): the results using Amp primer-probe; and (**C**): the results using CMVp primer-probe. The number of mice at each point is 17. The individual data are shown as a plot with bars indicating median and interquartile range. ND: not detected. *: *p* < 0.05 and **: *p* < 0.01 vs. the pre-values before the injection.

**Figure 6:**
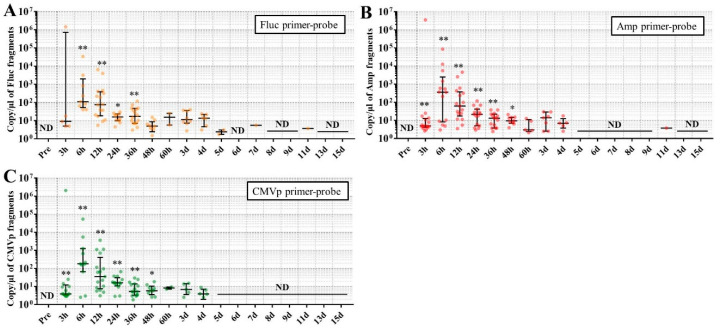
Long-term detection of multiple transgene fragments in stool DNA. (**A**): The results using Fluc primer-probe; (**B**): the results using Amp primer-probe; and (**C**): the results using CMVp primer-probe. The number of mice at each point is 17. The individual data are shown as a plot with bars representing median and interquartile range. ND: not detected. *: *p* < 0.05 and **: *p* < 0.01 vs. pre-values before the injection.

In experiment 3, the mice injected with pFluc-PEI complex and not sacrificed during the experiment were subjected to chronic detection of multiple transgene fragments using DNA from one drop of whole blood and DNA from stools. The results showed that the multiple transgene fragments were significantly detected for the blood-derived DNA until 6 days for Fluc primer-probe, 11 days for Amp primer-probe, and 9 days for CMVp primer-probe (Figure 5), compared with the pre-values before the injection. On the other hand, for the stool-derived DNA, the transgene fragments were also detected until 36 h for Fluc primer-probe and 48 h for Amp and CMVp primers-probes (Figure 6).

### 3.4. The Primers and TaqMan Probes Are Specific to the Targeted Transgene Fragment

**Figure 7:**
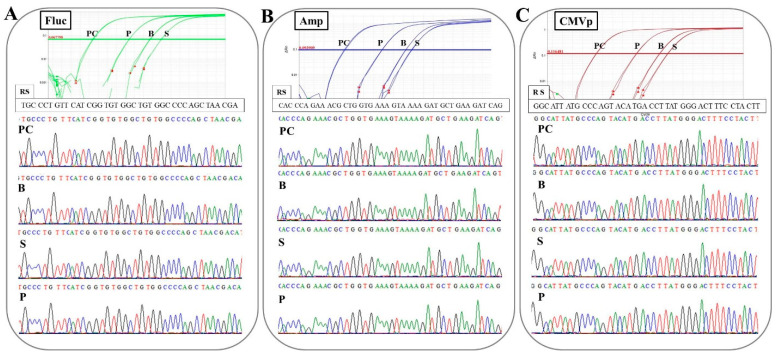
Determining the specificity of the primers-probes in this study. Upper panels display amplification plots of the TaqMan-qPCR and lower panels show partial waveforms and determined sequences from the Sanger sequencing for about 40 nucleotides. The sufficient amplifications and specific amplicons for targeted transgene fragments were confirmed in all primers-probes. The sequences of the amplicons also completely matched. (**A**): Using Fluc primer-probe, (**B**): using Amp primer-probe, and (**C**): using CMVp primer-probe. PC: positive control (100 pg/μL pFluc), B: blood-cell-fraction DNA, S: stool DNA, P: plasma cfDNA. RS: reference sequence. The correct pFluc sequence from the manufacturer’s (OXGENE, Oxford, UK) website was used as the reference sequence.

From the amplification plots of the TaqMan-qPCR assay, it was confirmed that the targeted transgene fragments of Fluc, Amp, and CMVp in the three specimens were fully amplified. From the results of Sanger sequencing, the waveforms to determine the nucleotides were accurate and all amplicons were completely matched over 90 or more bases to each reference sequence (Figure 7).

## 4. Discussion

In this study, in experiment 1, we first developed gene delivery model mice using pFluc with PEI, as a mimic of gene doping. The results showed that luminescence was fully confirmed on an in vivo imaging system in G4 (PEI + pFluc). In G2 (DW + pFluc), which was set as a control to check the effect of PEI, little luminescence emission was confirmed. Upon comparing the luminescence levels between G2 and G4, there was a greater than 10-fold difference from 6 h to 3 days. These results indicate that the plasmids were fully delivered to the cells in the abdominal cavity to induce exogenous gene expression when pFluc with PEI was injected. It was also indicated that a small amount of gene delivery was achieved even when the plasmid alone was injected. Taking these findings together, it can be considered that G4 is a better gene delivery model to mimic gene doping than G2. However, in this experiment, it was not clarified which tissue in the abdominal cavity took up the plasmid. Upon in vivo imaging, luminescence was observed throughout the abdomen below the diaphragm, so the plasmid could have been taken up by a range of abdominal organs and tissues such as epididymal fat, small and large intestine, mesentery, and fascia. It is thus possible that, using this method, abdominal organs could become new endocrine organs producing hormones of candidate genes such as erythropoietin (EPO), insulin-like growth factor 1 (IGF-1) and follistatin (FST) for gene doping, if the gene expressed by the plasmid is changed. Additionally, if researchers use this method, gene doping models could easily be established and further research on gene doping would accelerate.

In some studies on gene doping using animals injected with plasmids, the gene transfer efficiency into the targeted tissues was not measured [16,17,18]. Generally, when transfection is performed using plasmid alone, gene transfer efficiency is very low. This was also demonstrated in this study (Figure 2, G2: injected with plasmid alone). Therefore, it is unclear whether gene delivery into cells was actually sufficient in the aforementioned studies. To create an appropriate gene doping model, it would be necessary to measure the transfer efficiency of the gene of interest into targeted tissue. Some reports have described that the gene transfer efficiency into targeted tissues in gene doping model mice was measured by quantifying targeted protein and mRNA levels using Western blotting and qPCR [19,20]. As in these studies, measuring the gene or protein expression of the target would be more appropriate when creating a gene doping model in animals. However, this approach requires sacrificing the animals and is extremely laborious and time-consuming. In contrast, if the reporter signals are instead quantified using the luciferase gene in a gene doping study as in this study, the expression level of the transgene can be quantified in a short time without sacrificing the animal. Therefore, the delivery of a reporter gene such as luciferase would be a reasonable method when the researcher has selected a gene doping model.

In experiment 2, we aimed to detect multiple transgene fragments using TaqMan-qPCR assay. We explored which specimen had the highest sensitivity to detect the transgene fragments. The results showed that the three transgene fragments of Fluc, Amp, and CMVp were detected in G2 (DW + pFluc) and G4 (PEI + pFluc) in each specimen, with G4 in particular including a high level of the fragments. Upon comparing specimens of blood-cell-fraction DNA, stool DNA, and plasma cfDNA in G4 alone, the plasma cfDNA included the highest levels of the three transgene fragments, and was the specimen with the highest sensitivity; nevertheless, the total DNA concentrations were lowest among the three specimens. In general, a very low concentration of plasma cfDNA can be extracted from 200 μL of plasma, and it is almost impossible to measure the concentration with a spectrophotometer at a wavelength of 260 nm; this was confirmed by the results in this study and our previous study [20]. Conversely, plasma cfDNA had the most transgene fragments in this study, which is the opposite result from our previous study using adenoviral (AdV) vector [20]. Therefore, PEI and plasmid complexes would bind to plasma proteins and be stabilized after entering the blood vessels. Considering these results, plasma cfDNA could be the specimens providing the highest sensitivity for examining human gene doping with a plasmid with PEI. Additionally, Athlete Biological Passports (ABP) have been used to monitor selected biological variables in blood and urine over time, which can directly or indirectly reveal doping [21]. Considering the results of the experiment, examination of the gene fragments in plasma specimens as ABP could be one of the methods to detect gene doping using plasmid-PEI complex in athletes. Moreover, it could also be traceable from the early to the late stages of gene doping. However, if the vector type is changed to another vector such as AdV or adeno-associated virus (AAV) for gene doping, the specimens conferring the highest sensitivity might also change.

Using the stool DNA, multiple transgene fragments were detected in G2 (DW + pFluc) and G4 (PEI + pFluc). The stool is thus considered to be a particularly suitable specimen that can be obtained non-invasively. However, in G4, which is appropriate as a gene delivery model in this study, the stool DNA had the lowest sensitivity to detect the transgene fragments among the three specimens, which indicates that the PEI-plasmid complex is unstable in stool within the intestine. It is also considered that, in the intestine and stool, deoxyribo nuclease (DNase) activities with intestinal bacteria are higher than in whole blood. Therefore, the transgene fragments may have been rapidly degraded by the DNase in the intestine. If a method to increase the sensitivity of detection is developed, stool could be more suitable as a non-invasively obtained specimen for detecting gene doping in humans.

Considering the results in experiment 2, it was hypothesized that just a single drop of whole blood could be a highly sensitive and easily collected specimen for detecting multiple transgene fragments in the model injected with pFluc-PEI complex because the whole blood includes blood cells and the plasma fraction. Therefore, in experiment 3, we performed long-term detection of the transgene fragments and explored the temporal detection limit in each mouse using just one drop of whole blood without sacrifice. Simultaneously, we also collected the stools as non-invasively obtained specimens. The results showed that the multiple transgene fragments for Fluc, Amp, and CMVp were detected in the blood and stool DNA at many time points. In the statistical analysis, significant detection of the three transgene fragments was confirmed until 6 to 11 days in the blood-derived DNA, depending on the primer-probe. On the other hand, the temporal detection limit for the stool DNA was 36 to 48 h. There was a clear difference in these periods. Therefore, it was suggested that plasmid-PEI complex is more unstable in stool than in blood. Since these considerations are possible in experiment 2 as well, it can again be inferred that DNA of the transgene fragments in the stool is easily degraded.

Additionally, in experiment 3, the days during which significant detection of the transgene fragments in one drop of whole blood could be achieved were 6, 11, and 9 days for Fluc, Amp, and CMPp primer-probes, respectively. This appeared to depend on the characteristics of each primer-probe or targeted DNA sequences. There were no significant differences among Tm values of the primer-probes and the GC% of the amplicons in this study. On the other hand, there was a slight difference among the amplicon sizes, with the shortest amplicons being obtained in association with the Amp primer-probe. Therefore, it is possible that the detection efficiency depends on the size of the amplicon when low copy numbers of the fragments were detected. In addition, when designing a primer-probe for examination of gene doping, it could be necessary to pay attention to the length of the amplicon and to create several primer-probes to confirm the detection efficiency when the target fragments have a low copy number.

We previously reported results of another study similar to this one. This previous study showed that, when only naked plasmid of human EPO (hEPO) with CMV promoter was injected into the abdominal cavity of mice, the transgene fragments of the hEPO were significantly detected at a median of 9.18 copies/μL in whole blood (about one drop) at 48 h after the injection [18]. On the other hand, in this study, the transgene fragments of Fluc, Amp, and CMVp were significantly detected at medians of 97.5, 99.9, and 77.8 copies/μL in whole blood (about one drop) at 48 h from the injection of the plasmid-PEI complex. At 48 h in both studies, there was a large difference in the number of fragments, suggesting that mice injected with plasmid-PEI complex have more transgene fragments in the blood than those with only plasmid injection. Supporting this, in experiment 2 in this study, the number of transgene fragments was significantly higher in the group administered plasmid-PEI complex than in the group administered plasmid only. Taking these findings together, it is suggested that the plasmid-PEI complex is more stable in blood than plasmid alone, which could be a useful finding for gene doping research.

If the data are considered for qualitative analysis, some mice still had positivity for the detection of transgene fragments at 15 days using their primer-probes. It is generally considered that the turnover of metabolites occurs more rapidly in mouse than in humans. As described in the literature on drug metabolism in small animals, their metabolic rate is more than 10-fold higher than in humans [22,23]. Therefore, extrapolating these results to a human context, proof of gene doping in some cases could be detected using just a single drop of whole blood for a longer time after injection of the plasmid than in mouse. The use of a less invasive method such as fingertip blood sampling would also be recommended if the examination is performed on humans.

In the experiment on determining the specificity of primers-probes in this study, it was confirmed that the correct targeted sequences were accurately amplified in the qPCR with the combinations of the three primers-probes and three specimens. Additionally, there was no non-specific amplification or contamination in the control groups (G1 and G3; experiment 2), or at the pre-point before the injection (experiment 3) or in negative control wells. These results mean that the DNA extraction methods, primer-probe design, thermal cycle conditions, and reagents for TaqMan-qPCR assay in this study are accurate and appropriate. Therefore, the protocols constructed in this study could be directly applied to the examination of human samples. However, high-throughput assays based on these protocols may have to be established in the future. In this study, one primer pair was used per run. The use of multiplex-TaqMan-qPCR (mqPCR) assay has already been investigated in studies of a gene doping model in horses [24]. In high-throughput assays for examining human gene doping, mqPCR assay could be a very valuable tool because there is a risk of various genes and vectors being used for gene doping. In addition, as shown in this study, the detection of vector-specific sequences, such as CMV, CAG (combination of the CMV early enhancer element and chicken β-actin promoter), PGK (mouse phosphoglycerate kinase 1), GAPDH (human glyceraldehyde-3-phosphate dehydrogenase), and EF1α (human elongation factor 1 α) promoter, ampicillin and kanamycin resistance genes, and multi-cloning sites, as well as transgene sequences, could be important to confirm exogenous gene delivery, which could provide more robust proof of gene doping. In the future, we will create a multiplex PCR panel targeting the genes and vector-specific sequences for application to examine gene doping in athletes.

When creating qPCR primer-probes for genes with a risk of being exploited for gene doping, such as hEPO, hFST, and hIGF1, some ingenuity is required. This means that the target sequences must include the exon–exon junction. This is to avoid amplification from genes in the genome and maintain specificity to the vector. The length that can be extended by qPCR is generally about 500 bp. In contrast, because introns have a very large number of base pairs, amplification for exon-exon junctions is difficult in qPCR. On the other hand, the gene body inserted into a vector for gene delivery generally does not contain introns. Therefore, if a primer-probe is designed on an exon-exon junction, amplification can be expected to be transgene-specific. These findings have been clarified in previous studies [18,24,25]. However, this specific PCR amplification could be avoided by codon modifications of the gene body. When considering the codon modifications, analysis with next-generation sequencing (NGS) could be a useful tool. Therefore, we have undertaken research and development to apply NGS analysis to the examination of gene doping in athletes.

## 5. Conclusions

**Figure 8:**
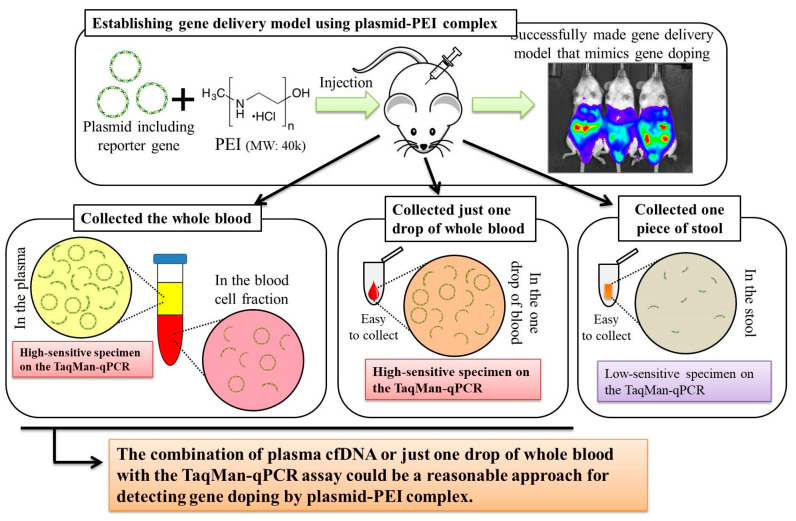
Summary of this study.

In this study, we aimed to develop a method to detect multiple transgene fragments as proof of plasmid-PEI-based gene doping. Here, the final summary is shown in Figure 8. Taking the findings together, our study showed that there was an abundance of multiple transgene fragments in plasma cfDNA in the plasmid-PEI-based gene delivery model mimicking gene doping. Moreover, it was suggested that the use of just a single drop of whole blood or plasma cfDNA with TaqMan-qPCR assay could be a reasonable method for examining plasmid-PEI-based gene doping in humans. Our new knowledge obtained in this study could accelerate the development of non-invasive methods of detecting gene doping in humans.

## Figures and Tables

**Table 1:** List of sequences of primers and TaqMan probes in this study.

Target		Sequence	Tm(°C)	Predicted Size(bp)	% Content of Amplicon
Fluc	Forward Probe Reverse	ATCGGATCGTGGTGTGTAGC	59.90	263	53
		56-FAM/TGCTGAACA/ZEN/GCATGGGCATCA/3IABkFQ	62.95		
		GCTTTGGAAGCCCTGGTAG	58.13		
Amp	Forward Probe Reverse	ACATTTCCGTGTCGCCCTTA	59.68	126	48
		56-FAM/ACCCAGAAA/ZEN/CGCTGGTGAAAGTAA/3IABkFQ	62.64		
		TCGATGTAACCCACTCGTGC	60.11		
CMVp	Forward Probe Reverse	TCATATGCCAAGTACGCCCC	59.89	188	49
		56-FAM/TGGGACTTT/ZEN/CCTACTTGGCAGTAC/3IABkFQ	62.08		
		CCCGTGAGTCAAACCGCTAT	60.11		

Fluc: firefly luciferase gene, Amp: ampicillin resistance gene, CMVp: cytomegalovirus promoter. Tm: melting temperature, GC: guanine and cytosine.
